# Miniscope Processing Suite (MPS): An Intuitive, No-Code, Scalable Pipeline for Long-Duration Calcium Imaging

**DOI:** 10.64898/2025.12.01.691517

**Published:** 2025-12-02

**Authors:** Ari Peden-Asarch, Meredith Weinstock, Kevin R. Coffey, John F. Neumaier

## Abstract

Miniscope calcium imaging provides a unique window into the activity of neurons during behavior while enabling spatial localization of individual cells across time. Despite its potential to revolutionize *in vivo* imaging alongside the rise of optogenetic tools, miniscopes remain underutilized. This gap may stem from the lack of an easy-to-use preprocessing software tailored to miniscope data. To address this need, we developed the first no-code end-to-end scalable pipeline for preprocessing large miniscope recordings: the Miniscope Processing Suite (MPS). MPS is the first implementation of Constrained Non-negative Matrix Factorization (CNMF) with Nonnegative Double Singular Value Decomposition (NNDSVD) initialization, multi-lasso segmentation, and parallelized temporal and spatial updates, enabling analysis of recordings exceeding three hours on a standard hardware. We tested a large dataset consisting of 28 operant behavior sessions (77 hrs, 7.26 TB of video data) on a single workstation. MPS completed the analysis in 55.6 hrs, (averaging 0.72 minute of processing time per minute of recording), which is 10-20X faster than traditional pipelines. Packaged as an easy downloadable plug-and-play software with a graphical user interface and requiring no coding or Git experience, MPS lowers the barrier to miniscope analysis while improving on current methodologies.

## Full Text Availability

The license terms selected by the author(s) for this preprint version do not permit archiving in PMC. The full text is available from the preprint server.

